# Надпочечниковая недостаточность в структуре Х-сцепленной адренолейкодистрофии

**DOI:** 10.14341/probl13335

**Published:** 2023-12-05

**Authors:** С. Р. Еникеева, И. С. Чугунов, М. А. Карева, М. В. Куркина, Е. Ю. Захарова, С. В. Михайлова, О. Б. Безлепкина, В. А. Петеркова, Н. Г. Мокрышева

**Affiliations:** Национальный медицинский исследовательский центр эндокринологии; Национальный медицинский исследовательский центр эндокринологии; Национальный медицинский исследовательский центр эндокринологии; Медико-генетический научный центр им. акад. Н.П. Бочкова; Медико-генетический научный центр им. акад. Н.П. Бочкова; Российский национальный исследовательский медицинский университет им. Н.И. Пирогова; Национальный медицинский исследовательский центр эндокринологии; Национальный медицинский исследовательский центр эндокринологии; Национальный медицинский исследовательский центр эндокринологии

**Keywords:** надпочечниковая недостаточность, Х-сцепленная адренолейкодистрофия, адреномиелонейропатия, церебральная форма, заместительная гормональная терапия, гидрокортизон, флудрокортизон

## Abstract

**ОБОСНОВАНИЕ:**

ОБОСНОВАНИЕ. Х-сцепленная адренолейкодистрофия (Х-АЛД) — тяжелое нейродегенеративное заболевание, встречается с частотой 1:17000 новорожденных мальчиков. Надпочечниковая недостаточность (НН), входящая в структуру Х-АЛД и встречающаяся у 70–80% пациентов, является жизнеугрожающим состоянием без своевременно назначенного лечения. Прогрессирующий характер адренолейкодистрофии, возможность присоединения НН в течение всего периода заболевания, отсутствие предиктивного фактора развития эндокринных нарушений диктует необходимость всестороннего изучения надпочечниковой недостаточности при данном заболевании.

**ЦЕЛЬ:**

ЦЕЛЬ. Изучить особенности диагностики и лечения надпочечниковой недостаточности при Х-АЛД.

**МАТЕРИАЛЫ И МЕТОДЫ:**

МАТЕРИАЛЫ И МЕТОДЫ. Ретроспективное наблюдательное сравнительное исследование диагностики и течения заболевания проведено у 66 пациентов мужского пола с генетически подтвержденным диагнозом «Х-сцепленная адренолейкодистрофия» и имевших надпочечниковую недостаточность как компонент Х-АЛД, находившихся на обследовании и лечении в Институте детской эндокринологии ФГБУ «НМИЦ эндокринологии» Минздрава России, МГНЦ им. акад. Н.П. Бочкова, РДКБ ФГАОУ ВО «РНИМУ им. Н.И. Пирогова» в 2010–2022 гг.

**РЕЗУЛЬТАТЫ:**

РЕЗУЛЬТАТЫ. Медиана возраста манифестации Х-АЛД составила 6,6 года [4,7; 11,1]. Самый ранний возраст установления диагноза НН — 1,5 года на доклинической стадии и 1 год 8 месяцев с клиническими проявлениями. Наследственный анамнез отягощен у 39,4% пациентов, у 15,1% (10/66 пациентов) заболевание установлено на доклинической стадии. У 59,1% (n=66) установлена церебральная форма заболевания (цАЛД), у 16,6% адреномиелонейропатия (АМН), у 24,2% изолированная надпочечниковая недостаточность (ИНН). Возраст установления НН в группе пациентов с АМН (15,6 года) статистически значимо отличался от установления НН у пациентов с цАЛД (7,4 года, р=0,001) и ИНН (5,6 года, р=0,000). Уровень ренина исследован у 22,7% при манифестации НН (15/66 пациентов), дефицит минералкортикоидов был установлен у 7 человек. Минералокортикоидную терапию назначали одновременно с глюкокортикоидной у пациентов с цАЛД, у пациентов с АМН и ИНН добавляли к терапии через 11 и 7 месяцев соответственно (различия между пациентами в группах с АМН и ИНН статистически не значимы). Комбинированную заместительную терапию получает 41% пациентов с цАЛД, 54,5% пациентов с АМН, 60% пациентов с ИНН.

**ЗАКЛЮЧЕНИЕ:**

ЗАКЛЮЧЕНИЕ. Все пациенты с установленным диагнозом Х-АЛД должны находиться под наблюдением эндокринолога на протяжении всей жизни для своевременной диагностики надпочечниковой недостаточности. Важно оценивать уровень ренина при манифестации НН и при динамическом наблюдении для диагностики минералокортикоидной недостаточности и назначения терапии.

Для исключения Х-АЛД необходимо обследовать всех пациентов мужского пола с надпочечниковой недостаточностью независимо от возраста манифестации.

Необходимо тщательное обследование родственников пациентов для выявления Х-АЛД на доклиническом этапе.

## Abstract (en)

**BACKGROUND:**

BACKGROUND: X-linked adrenoleukodystrophy (X-ALD) is a severe neurodegenerative metabolic disease with a frequency 1:17,000 in newborn boys. Being a major part of X-ALD with an incidence of 70–80% of patients, adrenal insufficiency (AI) is a life-threatening condition without timely treatment. The possibility of developing AI during the whole disease duration and the absence of any predictive factor for AI joining shows the necessity of studying AI in X-ALD patients to optimize current diagnostic and treatment algorithms.

**AIM:**

AIM: To study diagnostic and therapeutic features of primary adrenal insufficiency due to X-ALD.

**MATERIALS AND METHODS:**

MATERIALS AND METHODS: A retrospective observational comparative study was conducted in 66 male patients, examined and treated in the Pediatric endocrinology department of Endocrinology Research Centre, Research Centre for Medical Genetics, Research and Clinical Institute for Pediatrics of the Pirogov Russian National Research Medical University Detached Structural Unit Russian Children’s Clinical Hospital (Moscow, Russia) for 2014–2022. All of patients were diagnosed with primary AI and a genetically confirmed X-ALD.

**RESULTS:**

RESULTS: The median age of X-ALD manifestation was 6.6 years [4.7; 11.1]. The earliest age of AI diagnosis was 1.5 years at the preclinical stage and 1 year 8 months with clinical symptoms. The renin level was studied in 22.7% at the manifestation of AI (15/66 patients), mineralocorticoid deficiency was found in 7 patients. Family history was positive in 39.4% of patients (n=66), only in 15.1% (10/66 patients) of patients the disease was established at the preclinical stage. In 59.1% (n=66) the cerebral form of the disease (cALD) was established, in 16.6% — adrenomyeloneuropathy (AMN), and in 24.2% — isolated adrenal insufficiency (PAI). Age of AI establishment in the group of patients with AMN (15.6 years) significantly differs from the establishment of AI in patients with cALD (7.4 years, p=0.001) and PAI (5.6 years, p = 0.000). Mineralocorticoid therapy was prescribed simultaneously with glucocorticoid therapy in patients with cALD, in AMN and PAI patients it was added after 11 and 7 months, respectively (the differences between AMN and PAI groups were insignificant). Combined hormonal therapy receive 41% of patients with cALD, 54.5% of patients with AMN and 60% of patients with PAI.

**CONCLUSION:**

CONCLUSION: It is necessary to examine all male patients with AI regardless of the manifestation age to exclude adrenoleukodystrophy, and it is also important to examine patients for the presence of AI regardless of X-ALD manifestation age. The assessment of renin level in the manifestation of AI is also needed to prescribe mineralcorticoid therapy timely. Studying family history is the main method to detect X-ALD at the preclinical stage.

## Обоснование

Х-сцепленная адренолейкодистрофия (Х-АЛД) — нейродегенеративное заболевание, имеющее Х-сцепленный рецессивный тип наследования и характеризующееся вовлечением в патологический процесс нервной и эндокринной систем [1].

В 1910 г. учеными Haberfeld and Spieler было представлено первое описание клинического случая Х-АЛД у мальчика с развившейся надпочечниковой недостаточностью в возрасте трех лет с последующим присоединением неврологических нарушений в виде общей расторможенности, потери речи и способности к самостоятельному передвижению к 6,5 года, летальным исходом в возрасте 7 лет. Последующие описания серии клинических случаев были сделаны Schilder в 1912, 1913, 1924 гг., и адренолейкодистрофия некоторое время была известна как «болезнь Шильдера». Х-сцепленный характер наследования был определен в 1963 г. Fanconi et al. на основании описанных к этому времени клинических случаев. Термин «адренолейкодистрофия» был введен Michael Blaw в 1970 г. [2].

В России алгоритм биохимической (исследование уровня жирных кислот с очень длинной цепью — ОДЦЖК) и молекулярно-генетической (исследование гена ABCD1) диагностики адренолейкодистрофии впервые оптимизирован и введен в клиническую практику в МГНЦ им. акад. Н.П. Бочкова [3]. Описание неврологического дефицита при Х-АЛД, как одной из самых частых форм среди первичных лейкодистрофий, входящих в число наследственных болезней обмена веществ, и разработка алгоритмов дифференциальной диагностики с другими формами были проведены в ФГАУ ВО РНИМУ им. Пирогова в 2010 г. [4]. Тактика ведения пациентов мужского пола при выявлении первичной надпочечниковой недостаточности отражена в клинических рекомендациях и учебных пособиях, подготовленных ФГБУ «НМИЦ эндокринологии» [5][6].

В настоящее время проводятся исследования по оптимизации терапии церебральной формы заболевания [7][8], также продолжается изучение генофенотипической корреляции и поиск факторов, влияющих на клинический вариант заболевания и прогноз.

По данным литературы, адренолейкодистрофия встречается 1:17000 новорожденных мальчиков [9]. Х-АЛД является одной из самых частых патологий среди пероксисомных заболеваний и первичных лейкодистрофий у детей [4]. Доля Х-АЛД в структуре всей первичной надпочечниковой недостаточности у детей, по данным многоцентрового исследования, проведенного в Италии, составляет 3,1%, являясь второй по частоте патологией после врожденной дисфункции коры надпочечников наравне с аутоиммунным полигландулярным синдромом 1 типа [10].

Идея о введении адренолейкодистрофии в программу неонатального скрининга появилась в 2004 г. у Hugo Moser et al., но только к 2006-му подходящий маркер — С26:0-лизофосфатидилхолин (C26:0-лизоФХ) — был найден и предложен к использованию. В декабре 2013 г. адренолейкодистрофия введена в программу неонатального скрининга в штате Нью-Йорк (закон о введении Х-АЛД в программу скрининга носит название закона Эйдана — по имени мальчика, умершего от адренолейкодистрофии, родители которого внесли значимый вклад в популяризацию идеи неонатального скрининга в Нью-Йорке), к 2019 г. скрининг проводился в 14 штатах США [2], к 2022-му — в 24 штатах [11]. Неонатальный скрининг включает определение концентрации С26:0-лизоФХ в сухих пятнах крови методом жидкостной хроматографии тандемной масс-спектрометрии (LC-MS/MS), и в настоящее время пилотные проекты проводятся в небольшом количестве стран. По данным пилотных проектов, говорить об истинной частоте заболевания преждевременно, однако полученные результаты согласуются с общеизвестной частотой Х-АЛД [12].

Выделяют три основных формы заболевания: церебральную форму (цАЛД), адреномиелонейропатию (АМН), изолированную надпочечниковую недостаточность (ИНН) [2]. Известно, что в ходе естественного течения заболевания может произойти поражение центральной нервной системы, и тогда заболевание перейдет в другую, более тяжелую форму.

Тяжесть и высокая летальность при Х-АЛД обусловлены степенью поражения нервной системы. В настоящее время традиционным методом лечения церебральной формы заболевания является трансплантация гемопоэтических стволовых клеток, при которой основной сложностью является поиск донора и развитие посттрансплантационных осложнений [13]. Другим методом терапии является генотерапия на основе лентивирусного вектора, с помощью которого модифицируют собственные CD34+ клетки пациента, внедряя в них нормальный ген ABCD1 [14]. Основным ограничением этих двух методов является наличие узкого терапевтического окна — лечение возможно только на начальных стадиях болезни, которое часто бывает пропущено [13][14].

В раннем выявлении Х-АЛД в будущем важную роль будет играть неонатальный скрининг, но на данном этапе одним из главных методов ранней диагностики остается выявление пациентов на основании клинических симптомов и обследование родственников пациента.

Надпочечниковая недостаточность, входящая в состав эндокринных нарушений при Х-АЛД, является патологией, которая может быть одним из первых признаков этого заболевания и также являться причиной жизнеугрожающих состояний и даже смерти пациента без своевременно назначенного лечения. Важно отметить, что надпочечниковая недостаточность может быть как манифестным симптомом, так и присоединиться в течение всего периода заболевания. Отсутствие какого-либо предиктивного фактора развития эндокринных нарушений диктует необходимость пожизненного динамического наблюдения пациентов эндокринологом [1][2].

В основе патогенеза развития надпочечниковой недостаточности при Х-АЛД лежит токсическое разрушение стероидпродуцирующих клеток избыточно накапливающимися в них ОДЦЖК. Их накопление в надпочечниках начинается еще внутриутробно, однако процесс поражения развивается постепенно: сначала поражаются пучковая и сетчатая зоны, ответственные за синтез кортизола и надпочечниковых андрогенов, с дальнейшим распространением на клубочковую зону, ответственную за синтез альдостерона. Скорость распространения процесса и его интенсивность разная, поэтому отмечается высокая вариабельность возраста развития клиники надпочечниковой недостаточности [15].

Другим звеном патогенеза является встраивание жирных кислот с очень длинной цепью в мембраны стероидпродуцирующих клеток, приводящее к снижению синтеза гормонов вследствие снижения ответа на стимуляцию АКТГ [15].

Третьим звеном патогенеза является уменьшение уровня свободного холестерина, вследствие его связывания с ОДЦЖК; соответственно, он становится менее доступен для стероидпродуциующих клеток, и стероидогенез снижается. Чаще всего надпочечниковая недостаточность представлена изолированным дефицитом глюкокортикоидов, однако, согласно патогенезу, в некоторых случаях развивается сочетанный глюко- и минералокортикоидный дефицит [2][15].

## Цель ИССЛЕДОВАНИЯ

Изучить особенности диагностики, течения и лечения надпочечниковой недостаточности при Х-АЛД.

## МАТЕРИАЛЫ И МЕТОДЫ

## Место и время проведения исследования

Анализ диагностики и течения заболевания проведен у пациентов, находящихся на обследовании и лечении на базе Института детской эндокринологии ФГБУ «НМИЦ эндокринологии» Минздрава России, отделения медицинской генетики ОСП Российская детская клиническая больница ФГАОУВО «Российский национальный исследовательский медицинский университет им. Н.И. Пирогова» Министерства здравоохранения Российской Федерации и МГНЦ им. акад. Н.П. Бочкова за 09.2010–08.2022 гг.

## Изучаемая популяция

Пациенты мужского пола с генетически подтвержденным диагнозом «Х-сцепленная адренолейкодистрофия» и наличием надпочечниковой недостаточности как компонента Х-АЛД.

Критерии включения: наличие подтвержденного диагноза “Х-сцепленная адренолейкодистрофия” генетическим анализом (прямое секвенирование гена ABCD1), наличие надпочечниковой недостаточности по данным клинико-лабораторного обследования, подписание информированного согласия на участие в исследовании.

Критерии исключения: отказ от участия в исследовании.

## Способ формирования выборки из изучаемой популяции (или нескольких выборок из нескольких изучаемых популяций)

Сплошной способ формирования выборки.

## Дизайн исследования

Многоцентровое наблюдательное динамическое ретроспективное сравнительное исследование.

## Методы

Проведен анализ возраста манифестации Х-АЛД, времени от манифестации до присоединения дополнительного компонента заболевания. Манифестация заболевания может быть с клиники надпочечниковой недостаточности, с клиники поражения нервной системы, а также диагноз может быть установлен при обследовании родственников пациента. Пациенты были разделены на группы в соответствии с формой манифестации.

Далее пациенты были разделены на группы в соответствии с клинической формой заболевания.

Клиническая форма болезни определялась согласно международному консенсусу по ведению пациентов с Х-АЛД по совокупности данных исследования. Церебральная форма заболевания устанавливалась при наличии типичных очагов лейкодистрофии по данным МРТ головного мозга (гиперинтенсивный сигнал в Т2-взвешенном изображении и FLAIR режимах, снижение интенсивности сигнала в Т1-взвешенных изображениях в области мозолистого тела, кортикоспинальных и кортикопонтинных трактов, с возможным распространением в затылочные и задние теменные отделы). Адреномиелонейропатия устанавливалась в отсутствие поражения головного мозга по данным МРТ при наличии неврологических симптомов заболевания (нарушение походки, спастический парапарез, нарушение функции тазовых органов и т.д.). Изолированная надпочечниковая недостаточность устанавливалась при наличии соответствующих клинико-лабораторных признаков надпочечниковой недостаточности и отсутствии неврологических проявлений адренолейкодистрофии (клинически и по данным МРТ головного мозга).

Каждому пациенту проведено клиническое обследование (анамнез жизни и заболевания, исследование родословной), антропометрия (оценка роста, веса, полового развития по шкале Таннера). Дефицит или избыток роста и веса устанавливался при отклонении коэффициента стандартного отклонения на 2 SD от среднепопуляционных значений (SDS роста, для веса оценивался SDS индекса массы тела (ИМТ)), расчет производился по программе Auxology (референсные данные: UK Tanner Whitehouse).

Лабораторная диагностика включала исследование гормональных показателей (АКТГ, кортизол, ренин), биохимических показателей (натрий, калий, глюкоза сыворотки крови).

Показатели биохимического анализа крови определялись на автоматическом биохимическом анализаторе «Architect c8000» («Abbott Laboratories», США) по стандартным методикам с использованием реагентов производителя. Определение концентраций гормонов выполнялось иммунохемилюминесцентным методом на автоматизированной системе Cobas 600 (Roche, Франция).

Также всем пациентам была проведена МРТ головного мозга с внутривенным контрастированием. Магнитно-резонансная томография головы проводилось на аппарате «Magnetom Harmony» (Siemens, Германия) c напряженностью магнитного поля 3,0 Тесла, режим Т1, Т2, FLAIR с введением контрастного препарата на основе гадолиния.

Молекулярно-генетический анализ проводился методом прямого секвенирования гена ABCD1 в лаборатории селективного скрининга ФГБНУ МГНЦ им. акад. Н.П. Бочкова. Оценка патогенности вариантов нуклеотидной последовательности проводилась согласно международным и российским рекомендациям. Выделение ДНК из образцов крови осуществлено с помощью набора Diatom DNA Prep reagent kits (Biocom, Russia) при следовании рекомендациям производителя. Секвенирование гена ABCD1 произведено с использованием секвенатора ABI Prism 3500 (Applied Biosystems) при следовании протоколу производителя. Праймеры для проведения ПЦР синтезированы на основе референсной последовательности гена ABCD1 NM_ 000033.3.3.3.4 с включением всех экзонов и примыкающих интронных областей.

Диагноз надпочечниковой недостаточности устанавливался по совокупности клинических данных (слабость и вялость, гиперпигментация кожных покровов, эпизоды повторных рвот, тяга к соли) и данных лабораторного обследования. Дефицит глюкокортикоидных гормонов был установлен при получении двукратного превышения АКТГ верхней границы референсного интервала лаборатории (>150 пг/мл) при уровне кортизола менее 500 нмоль/л. Дефицит минералокортикоидных гормонов был установлен при получении показателей ренина, двукратно превышающих верхнюю границу референсного интервала (>100 мкЕд/мл) и/или гипонатриемии (натрий<135 ммоль/л) и гиперкалиемии (калий >5,1 ммоль/л).

## Статистический анализ

Расчет статистических показателей производился с помощью статистического пакета Statistica 12 (StatSoft inc., США). Описательная статистика количественных признаков представлена медианами и 25-м и 75-м квартилями. Описательная статистика качественных признаков представлена абсолютными и относительными частотами. Для сравнения двух независимых групп использован критерий Манна-Уитни, уровень р<0,05 считался статистически значимым. Для сравнения трех групп использован критерий Краскела-Уоллиса с поправкой Бонферрони на множественные сравнения. При выявлении статистически значимых различий проводилось попарное сравнение с использованием критерия Манна-Уитни.

## Этическая экспертиза

Проведение исследования одобрено локальным этическим комитетом ФГБУ «НМИЦ эндокринологии» Минздрава России. Протокол №24 от 24.11.2021 г.

## Результаты

В исследование включено 66 пациентов с доказанными патогенными вариантами гена ABCD1 и подтвержденным диагнозом надпочечниковой недостаточности (НН). Медиана длительности динамического наблюдения составила 3,65 года [ 2,03; 7,3], от 6 месяцев до 33 лет (из подсчета исключены впервые выявленные пациенты).

Среди обследованной группы пациентов (n=66) самыми частыми жалобами при первом обращении к врачу были слабость (35 человек, 53%), гиперпигментация кожных покровов (32 человека, 48,5%), эпизоды повторных рвот (25 человек, 37,9%). Среди первых симптомов болезни неврологического характера были нарушение походки (15 человек, 22,7%), нарушения эмоциональной сферы (13 человек, 19,7%), нарушения высших корковых функций (12 человек, 18,2%), снижение слуха (10 человек, 15,2%). Спектр жалоб при манифестации заболевания представлен на диаграмме 1.

**Figure:**
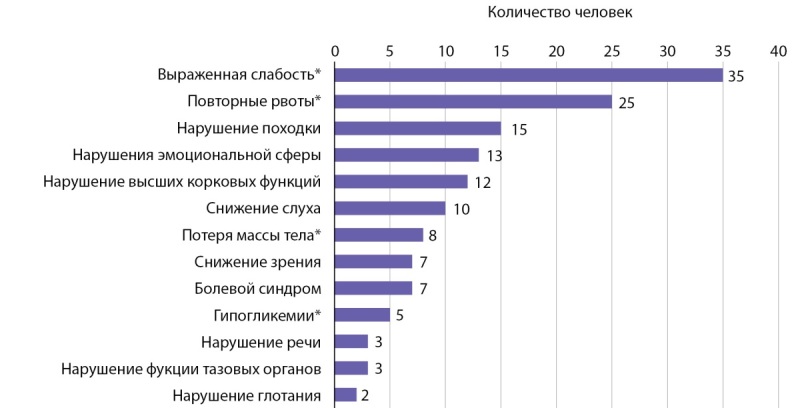
Диаграмма 1. Спектр жалоб при Х-АЛД. Примечание: на данной диаграмме представлены жалобы на момент манифестации заболевания. Общее число пациентов — 66 человек. Символом «*» отмечены жалобы, более характерные для надпочечниковой недостаточности.

Медиана возраста манифестации симптомов адренолейкодистрофии, независимо от варианта дебюта, среди всех обследованных пациентов (n=66) составила 6,6 года [ 4,7; 11,74]. До окончательного подтверждения диагноза проходило в среднем 2,3 года, и медиана возраста постановки диагноза «Х-сцепленная адренолейкодистрофия» составила 8,9 года [ 6,7; 13,9].

Среди всех пациентов (n=66) симптомы НН манифестировали в среднем в 6,7 года [ 5,0; 11,3]. Медиана возраста подтверждения диагноза «Надпочечниковая недостаточность» составила 8,3 года [ 6,3; 12,6]. Таким образом, диагноз «НН» был установлен в среднем через 1,6 года после манифестации. Среди тех пациентов, у которых НН стала первым симптомом заболевания, диагноз «Х-АЛД» был установлен в 8,9 года [ 6,7; 13,9].

Самый ранний возраст диагностики НН — 1,5 года, при обследовании двух пациентов в связи с отягощенным наследственным анамнезом, самый поздний возраст развития НН — 32 года (2 пациента), НН в данных случаях присоединилась к уже существующему поражению нервной системы. Самый ранний возраст манифестации клинических симптомов НН — 1 год 8 месяцев.

Манифестация с клиники НН отмечена у 40 человек (60,7%), клиника поражения нервной системы как первой причины заболевания была у 16 человек (24,2%). На доклинической стадии в связи с отягощенным наследственным анамнезом диагноз адренолейкодистрофии был установлен 10 пациентам (15,1%). Медиана возраста в зависимости от варианта манифестации, а также время до присоединения дополнительного компонента заболевания представлены в таблице 1.

**Table:** Таблица 1. Сравнительные характеристики групп пациентов в зависимости от варианта дебюта Х-сцепленной адренолейкодистрофии Примечание: НС — нервная система; НН — надпочечниковая недостаточность; НА — наследственный анамнез. Уровень p<0,025 считался статистически значимым с учетом поправки Бонферрони на множественные сравнения.

	Дебют с поражения НС	Дебют с клиники НН	Обследование по отягощенному НА	р, Краскела-Уоллиса ANOVA
Количество пациентов	16	40	10	-
Медиана возраста манифестации, лет	6,6 [ 5,5; 10,9]	7,0 [ 5,0; 11,3]	7 [ 3,6; 12,6]	0,622
Время до присоединения дополнительного компонента, лет	До присоединения НН 1,2 [ 0,7; 1,7]	До присоединения поражения НС 2,8 [ 1,5; 5,5]		0,051

Среди пациентов, обследованных на доклинической стадии заболевания, у 8 человек (80,0%) диагноз надпочечниковой недостаточности был установлен при первичном обследовании, у 2 пациентов отмечалось развитие НН через 2,1 и 2,5 года. У 5 человек из 10 впоследствии отмечено присоединение поражения головного мозга по данным МРТ, и одному была проведена трансплантация гемопоэтических стволовых клеток, двоим — генная терапия с помощью лентивирусного вектора в рамках клинического испытания препарата, 1 взрослый, который находится на динамическом наблюдении, и одному пациенту осуществляется поиск донора.

Всего наследственный анамнез отягощен у 26 человек (39,4% обследованных), однако диагноз «Х-АЛД» заподозрен и установлен на основании семейного анамнеза лишь 10 пациентам (15,1%).

При первичном лабораторном обследовании данные об определении уровня глюкозы крови имеются у 14 пациентов (21,2%), уровня электролитов (натрий, калий) у 24 пациентов (36,6%). Гипогликемия (уровень глюкозы крови менее 3,3 ммоль/л) отмечена у 5 пациентов (7,6%), а значимое снижение уровня натрия (менее 135 ммоль/л) выявлено у 16 пациентов (24,2%). Медиана уровня натрия при манифестации НН — 131,5 ммоль/л [ 122; 137,25]. Гиперкалиемия (выше 5,1 ммоль/л) отмечена у 7 человек (10,6%), максимальный уровень калия составил 6,2 ммоль/л, медиана уровня калия — 4,53 ммоль/л [ 4,03; 5,35]. Развитие кризовых состояний хотя бы один раз отмечено у 29 человек (43,9%).

При манифестации НН в гормональном анализе отмечалось значимое повышение уровня АКТГ до 1187 пг/мл [ 337,5; 1250,0] одновременно со значимым снижением уровня кортизола до 97,3 нмоль/л [ 44,5; 181,8], что подтверждало диагноз.

При манифестации НН гормональные исследования для подтверждения или исключения дефицита минералкортикоидов проведены у 15 человек (22,7%, n=66), у 7 из которых наличие минералокортикоидного дефицита подтверждено. При этом прямой ренин измерен у 10 человек — медиана уровня ренина составила 107,7 мЕд/л [ 74,25; 221,6], диагностически значимое повышение уровня ренина (>100 мЕд/л) — у 5 человек. У 5 человек измерена активность ренина плазмы (АРП), у 2 из которых отмечается значимое повышение (>10 нг/мл/час) — 39 и 339 нг/мл/час.

Рост и вес в представленной когорте пациентов не отличался от популяционного — медиана SDS роста 0,27 [ -0,48; 0,93], медиана SDS ИМТ — -0,03 [ -1,0; 0,46]. Низкие показатели роста и веса (SDS <2,0) отмечались всего у 5 пациентов с церебральной формой заболевания и у 2 пациентов с ИНН.

На момент обследования на комбинированной глюко- и минералокортикоидной заместительной терапии (ГК- и МК-терапия) находился 31 человек (46,9%, n=66). Медиана дозы гидрокортизона независимо от клинической формы Х-АЛД составила 12,2 мг/м²/сут [ 9,88; 14,85], флудрокортизона — 50 мкг/сут [ 46,9; 81,3].

Сравнительные характеристики групп пациентов по формам Х-АЛД (церебральная форма, адреномиелонейропатия, изолированная надпочечниковая недостаточность) представлены в таблице 2.

**Table:** Таблица 2. Сравнительные характеристики клинических форм Х-сцепленной адренолейкодистрофии Примечание: Х-АЛД — Х-сцепленная адренолейкодистрофия; НН — надпочечниковая недостаточность; ИМТ — индекс массы тела. Данные по возрасту манифестации Х-АЛД и установления НН представлены в виде медианных значений с указанием 25-го и 75-го квартилей. Уровень р<0,006 считался статистически значимым с учетом поправки Бонферрони на множественные сравнения. Р1-2 — коэффициент статистической значимости при попарном сравнении церебральной формы и адреномиелонейропатии, Р1-3 — коэффициент статистической значимости при попарном сравнении церебральной формы и изолированной надпочечниковой недостаточности, Р2-3 — коэффициент статистической значимости при попарном сравнении адреномиелонейропатии и изолированной надпочечниковой недостаточности.

	Церебральная форма	Адреномиело-нейропатия	Изолированная надпочечниковая недостаточность	р, Краскела-Уоллиса ANOVA
Количество пациентов	39	11	16	-
Возраст манифестации Х-АЛД	6,4 года [ 4,5; 9,1]	12,6 года [ 8,0; 18,5]	5,6 года [ 4,0; 8,5]	0,021
Возраст установления НН	7,4 года [ 6,2; 10,2]	15,6 года [ 12,0; 18,5]	7,5 года [ 5,9; 9,7]	0,0008 Р1-2 0,040 Р1-3 1,000 Р2-3 0,029
Медиана SDS роста	0,41 SD [ -0,76; 0,93]	0,24 SD [ -0,14; 0,63]	0,26 SD [ -0,28; 0,91]	0,959
Медиана SDS ИМТ	-0,38 SD [ -1,41; 1,38]	0,51 SD [ 0,0; 1,01]	-0,02 [ -0,46; 0,52]	0,089
Медиана дозы гидрокортизона, мг/м²/сут	12,2[ 10,5; 15,3]	13,9[ 11,8; 15,9]	10,9[ 9,4; 13,4]	0,534
Медиана дозы флудрокортизона, мкг/сут	50,0[ 46,9; 100,0]	62,5[ 50,0; 75,0]	62,5[ 40,6; 112,5]	0,936
Количество пациентов на минералокортикоидной терапии, %	16 (41%)	6 (54,5%)	9 (60%)	-
Медиана длительности наблюдения	2,7 года [ 1,6; 4,6]	6,4 года [ 4,3; 8,9]	6,1 года [ 4,6; 8,9]	0,079
Медиана времени между началом ГК и добавлением МК-терапии	0 лет [ 0; 0]	0,9 года (11 месяцев) [ 0,3; 2,0]	0,6 года (7 месяцев) [ 0,0; 1,9]	0,0009 Р1-2 0,005 Р1-3 0,036 Р2-3 1,000

## Обсуждение

Надпочечниковая недостаточность является одним из главных компонентов Х-сцепленной адренолейкодистрофии, встречается, по данным многих исследований, в 70–80% случаев[16–18].

Учитывая прогрессирующий характер заболевания, риск развития НН остается высоким в течение всей жизни с преимущественной манифестацией в первой ее декаде, что согласуется с полученными нами данными [16][17].

Возраст манифестации Х-АЛД и НН как компонента не отличался от возраста, описанного в литературе и у пациентов с церебральной формой и изолированной НН, — и Х-АЛД, и НН манифестировали на первой декаде жизни. Среди пациентов с адреномиелонейропатией заболевание манифестирует в более старшем возрасте, что также соответствует мировым наблюдениям [2].

В настоящее время считается, что развитие надпочечниковой недостаточности обычно происходит у детей старше трех лет, однако Huffnagel et al. и Dubey et al. в 2019 г. показали развитие НН у пациентов более младшего возраста — в возрасте 7 и 5 месяцев (на доклинической стадии) [16][17]. По данным нашего исследования, также выявлены случаи развития НН в более раннем возрасте — 1,5 года (на доклинической стадии) и 1 год 8 месяцев (с клиническими проявлениями). Наличие надпочечниковой недостаточности по результатам гормонального обследования вне клинических проявлений отмечается у 50–70% пациентов по данным литературы [16][17], что также согласуется с показателями проведенного нами исследования (НН выявлена у 80,0%). Таким образом, важно проводить скрининг на наличие НН даже при отсутствии клиники, независимо от возраста установления Х-АЛД.

Тщательный сбор наследственного анамнеза и обследование родственников приведет к возрастанию числа пациентов, выявленных на доклинической стадии заболевания, увеличивая таким образом возможность своевременного применения этиотропного и патогенетического лечения, а также уберегая от возникновения жизнеугрожающих состояний вследствие развития недиагностированной надпочечниковой недостаточности. У обследованной нами когорты пациентов, несмотря на большую долю пациентов с отягощенным наследственным анамнезом (39,4%, 26 человек), Х-АЛД установлена на доклинической стадии только у 10 человек (38,5%).

Сроки установления диагноза НН в нашем исследовании составляют в среднем 2–3 года от начала клинических проявлений [16]. Эти сроки зависят от многих причин, среди которых можно выделить степень выраженности клинической картины, настороженность врачей, а также различные подходы к комплексному обследованию пациентов с Х-АЛД. Диагноз адренолейкодистрофии, по данным нашего исследования, был установлен в среднем через 2,3 года, независимо от клиники манифестации, что является достаточно длительным сроком и ухудшает прогноз пациентов. Установление диагноза в течение 1 года считается ранней диагностикой, которая позволяет выполнять мониторинг состояния нервной системы пациента тщательнее и проводить этиотропную или патогенетическую терапию на ранних стадиях [19]. По данным зарубежной литературы, рекомендуется проведение обследования для исключения надпочечниковой недостаточности при Х-АЛД начиная с 6 месяцев жизни каждые 3–6 мес до 10 лет, и затем — ежегодно [1][16][20]. В связи с этим представляется важным создание российских клинических рекомендаций по ведению этой группы пациентов.

Первичная надпочечниковая недостаточность в основном обладает неспецифическими клиническими проявлениями, имеющими хронический характер. Среди всех ее симптомов [21] основными являются слабость и утомляемость, потеря массы тела, гипотония, боль в животе, рвота, задержка физического развития. Специфическим проявлением НН является гиперпигментация кожных покровов, выраженная тяга к соли (при наличии дефицита минералкортикоидов) [20]. Жизнеугрожающими проявлениями надпочечниковой недостаточности являются кризовые состояния, как вследствие электролитных нарушений, так и вследствие выраженной гипогликемии [22], — их характеризует выраженная слабость, нарушение сознания, выраженная гипотония [20].

Рост и вес в исследуемой когорте пациентов значимо не отличался от популяционного, так же как при распределении по группам в соответствии с клиническими формами заболевания. Единичные показатели, выходящие за рамки референсного интервала, отмечались в группах пациентов с церебральной формой заболевания и изолированной надпочечниковой недостаточностью, — это может быть связано с более тяжелым течением заболевания у пациентов с церебральной формой, а также, вероятно, с более ранним возрастом манифестации заболевания в группах с цАЛД и ИНН.

Согласно нашему исследованию и данным зарубежных авторов, монотерапия глюкокортикоидами, а также сочетанная глюко- и минералокортикоидная терапия назначались пациентам приблизительно в равном проценте случаев [16], что говорит в первую очередь о поражении пучковой зоны коры надпочечников при сохранении клубочковой зоны интактной. Наиболее чувствительным маркером наличия минералокортикоидной недостаточности является исследование уровня ренина крови. Однако при манифестации НН данный показатель исследуется не у всех пациентов, это может быть связано с отсутствием настороженности врачей, забором крови для исследования после стабилизации состояния на фоне массивной инфузионной терапии, а также другими техническими трудностями в проведении исследования. По данным нашего и зарубежных исследований, скрининг дефицита минералкортикоидов при манифестации НН проводится у малого количества пациентов (22,7% — по данным нашего исследования и 16,8% — по данным Huffnagel et. al in 2019), что приводит к гиподиагностике минералокортикоидного дефицита у пациентов с Х-АЛД [16].

Прогрессирующий характер поражения коры надпочечников диктует необходимость динамически оценивать функцию клубочковой зоны перед назначением флудрокортизона у пациентов с уже установленной надпочечниковой недостаточностью.

Интерес представляет отсутствие взаимосвязи между дозой гидрокортизона и наличием побочных эффектов терапии: средняя заместительная доза глюкокортикоидов по данным настоящего исследования больше, чем по данным исследования Huffnagel et al. от 2019 г. [16] (12,2 мг/м²/сут против 10,2 мг/м²/сут), однако осложнений в виде задержки роста, избытка веса у наблюдаемой нами когорты пациентов не выявлено. Оценка скорости роста как показателя отсутствия избытка глюкокортикоидной терапии, по данным зарубежной литературы и нашего исследования, не проводилась. Однако, учитывая отсутствие различий SDS роста и ИМТ от популяционных, по данным нашего исследования, вероятно, назначаемая доза ЗГТ не является избыточной для поддержания компенсации без развития побочных эффектов терапии.

Отмечаются различия и в средней дозе флудрокортизона, назначаемого пациентам: по данным нашего исследования, удовлетворительная компенсация достигнута на дозе 50 мкг/сут, по данным исследования Huffnagel et al. от 2019 г. — 100 мкг/сут [16]. Вероятно, это связано с различным подходом к ведению пациентов с Х-АЛД.

Выявленные различия в заместительной дозе гидрокортизона в зависимости от клинической формы заболевания (наибольшее количество гидрокортизона получают пациенты с АМН, наименьшее — с ИНН), вероятно, связаны с возрастом манифестации НН и разницей в подходах к лечению взрослых пациентов и детей.

Также одновременное назначение заместительной глюко- и минералокортикоидной терапии пациентам с церебральной формой вместо постепенного добавления минералокортикоидной в план лечения пациентам с АМН и ИНН может быть связано с тяжестью общего состояния, нежеланием оставлять пациента на динамическом наблюдении при получении лабораторных показателей в серой зоне, более внимательным подходом к скринингу компонентов адренолейкодистрофии.

## Ограничение исследования

В ходе исследования могли возникнуть смещения результатов из-за малого объема выборки в связи с редкой встречаемостью заболевания, а также исключения из исследования пациентов только с неврологическими нарушениями и отсутствием НН.

## Заключение

Надпочечниковая недостаточность как компонент Х-АЛД характеризуется постепенным прогрессированием заболевания в виде этапного вовлечения в патологический процесс зон коры надпочечников. Церебральная форма заболевания характеризуется ранним дебютом с одновременным развитием глюко- и минералокортикоидного дефицита. Для адреномиелонейропатии характерна более поздняя манифестация заболевания и этапное развитие глюко- и минералокортикоидного дефицита. ИНН занимает промежуточную позицию и, являясь больше диагнозом исключения, требует более внимательного наблюдения за появлением изменений, характерных для нервной системы при адренолейкодистрофии.

Обследование пациентов мужского пола с первичной надпочечниковой недостаточностью на наличие адренолейкодистрофии необходимо проводить независимо от возраста манифестации заболевания. Данные меры помогут улучшить и ускорить диагностику заболевания, тем самым увеличивая шансы пациентов получить эффективное лечение вовремя.

Необходимо проводить скрининг на наличие НН всем пациентам с установленным диагнозом «Х-АЛД» независимо от наличия симптомов и возраста манифестации, это уменьшит время диагностики НН и позволит вовремя назначить заместительную терапию.

У пациентов с наличием подтвержденной надпочечниковой недостаточности важно проводить оценку уровня ренина (активности ренина плазмы) для своевременного установления минералокортикоидного дефицита, назначения и коррекции соответствующей терапии. Это поможет предотвратить либо сократить количество кризовых состояний, представляющих угрозу жизни пациента.

Проведение скрининга в семьях, где выявлен пациент с Х-АЛД, в настоящее время является единственным способом установления диагноза Х-АЛД и НН как ее компонента на доклинической стадии. Он должен проводиться обязательно среди родственников мужского пола по материнской линии.

## Дополнительная информация

Источники финансирования. Работа выполнена по инициативе авторов без привлечения дополнительного финансирования.

Конфликт интересов. Авторы декларируют отсутствие явных и потенциальных конфликтов интересов, связанных с содержанием настоящей статьи.

Участие авторов. Еникеева С.Р., Чугунов И.С., Карева М.А. — поисково-аналитическая работа и подготовка финальной версии статьи; Безлепкина О.Б., Петеркова В.А., Мокрышева Н.Г. — идея и дизайн исследования, редактирование текста, внесение ценных замечаний; Куркина М.В., Захарова Е.Ю. — проведение генетического исследования, интерпретация результатов, редактирование текста и внесение ценных замечаний; Михайлова С.В. — клиническое обследование пациентов, редактирование текста, внесение ценных замечаний. Все авторы одобрили финальную версию статьи перед публикацией, выразили согласие нести ответственность за все аспекты работы, подразумевающую надлежащее изучение и решение вопросов, связанных с точностью или добросовестностью любой части работы.
